# Growth of High Aspect Ratio Wurtzite GaAs Nanowires

**DOI:** 10.1021/acs.cgd.5c00312

**Published:** 2025-08-22

**Authors:** M. M. Jansen, W. H. J. Peeters, D. Lamon, M. F. Schouten, M. A. Verheijen, E. P. A. M. Bakkers

**Affiliations:** † Department of Applied Physics, 3169 Eindhoven University of Technology, Groene Loper 19, Eindhoven 5612AP, The Netherlands; ‡ Eurofins Materials Science BV, High Tech Campus 11, Eindhoven 5656, The Netherlands

## Abstract

Crystal phase control of III–V semiconductor nanowires grown by the vapor liquid solid mechanism has emerged as a new frontier in nanomaterials in the 2010s. Of particular interest is the ability to grow the metastable wurtzite crystal, which is commercially unavailable in semiconductors such as GaAs and SiGe. The successful growth of wurtzite GaAs nanowires has been demonstrated by precise control of the wetting contact angle of the catalyst particle. However, a recent discovery revealed an inherent limitation, known as the critical length, which restricts the maximum achievable aspect, length-to-diameter, ratio in wurtzite GaAs nanowire below 100. Here, we demonstrate the growth of wurtzite GaAs nanowire above the cirtical length with a stacking fault density of 10 SF/μm and precise crystal phase control down to the monolayer regime using Ga-pulses. The crystal phase control by Ga-pulsing is investigated as a function of pulse duration, frequency and position along the nanowire length. A pulse scheme is developed to stabilize the wurtzite crystal phase for aspect ratios up to nearly 200. This method, involving controlled transitions between wurtzite and zinc blende phases, expands the potential of the GaAs platform to create superlattices in high aspect ratio nanowires.

## Graphical abstract

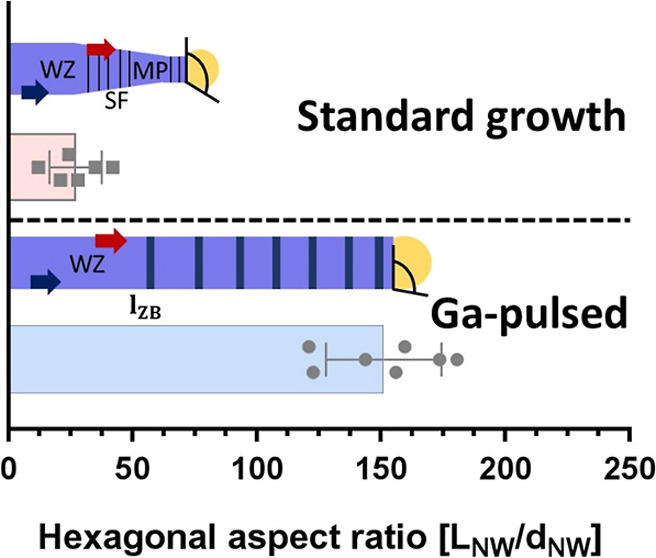

## Introduction

III–V nanowires (NWs) have emerged as a prominent semiconductor platform for nanophotonic devices such as detectors and lasing cavities.^1,2^ Thereby, radial heterostructures have been investigated utilizing the unique flexibility in strain compensation^3^ and crystal phase selection^4–12^ of the NW template enabling completely new heterostructure systems. The crystal structure of NWs not only influences their mechanical, thermal, and chemical behavior, but also governs their electronic and optical properties, offering unprecedented opportunities for tailoring semiconductor properties and functionalities.^13^

Recently, III–V NWs, such as GaP and GaAs, served as templates for the growth of metastable hexagonal (hex) SiGe.^11,14^ Hex-SiGe is of particular interest for the fabrication of Si-based laser cavities, which are a key milestone for silicon photonics.^15,16^ However, the lasing capabilities of the core/shell structures are highly dependent on the wurtzite (WZ) GaAs template quality.^17^ High-aspect-ratio NWs with thin cores and highly uniform facets are anticipated to reduce strain effects and promote consistent growth dynamics.^12,18^

Researchers have placed particular emphasis on unraveling the primary mechanism behind the phase selection mechanism in GaAs NWs grown via the vapor liquid solid (VLS) mechanism. In the VLS mechanism, a liquid catalyst particle is supersatured with atoms from the gas phase resulting in the crystallization of NWs. Various research groups, including Glas et al.,^4^ Dubrovski et al.,^19^ Jacobsson et al.,^20^ and Panciera et al.,^21^ investigated phase change mechanisms both theoretically and using in situ transmission electron microscopy (TEM). The contact angle between the catalyst particle and the NW emerged as the critical parameter for phase control, with the WZ phase nucleated at angles between 90° and 120°.^20^ Initial phase control on a wafer scale was achieved by Joyce et al.^22^ and Lehman et al.^23^ using MOVPE techniques, later complemented by molecular beam epitaxy techniques developed by Jansen et al.^24^ Despite the above-mentioned advancements, achieving high aspect, length-to-diameter, ratio above 100 with crystal phase control over extended lengths remained elusive. An overview of recent wurtzite GaAs growth studies is given in Figure 1. A recent discovery revealed an inherent limit to high aspect ratios WZ GaAs NWs known as the critical length (*L*_Cr_).^25^ The critical length is determined by the NW diameter and is defined as the NW length at which polytypism begins to occur in otherwise WZ GaAs NWs. It is hypothesized that the crystal phase switch is caused by As adatom diffusion along the side facets and edges of the NW, leading to the reduction of the contact angle below 90°.^25^ We stress that this is a hypothesized mechanism based on observed growth behavior.^25^ Given the sensitivity of WZ GaAs growth to the local V/III ratio, the low As supply (V/III ratio of 2.4) and the unprecedentend NW length achieved (Figure 1a), we propose that As surface diffusion may play a relevant role under these specific growth conditions. Despite being generally considered insignificant,^7,8,24,26^ the diffusion of volatile group V species continues to raise questions, as it may account for observations in which its influence cannot be ruled out.^25,27,28^ Ref 25 systematically evaluates and dismisses several factors that might contribute to contact angle reduction and hence critical length formation, including changes in the V/III ratio, growth temperature, substrate interactions, and Gibbs–Thomson effects. Moreover, consistent with previous reports,^20,29^ no Au incorporation from the Au catalyst into the GaAs NW is observed by APT.^18^ The critical length specifically limits the growth of thin (≤60 nm) GaAs NWs to aspect ratios of ≤30, which are expected to obtain the highest potential to relax strain induced by a lattice mismatched shell elastically due to their high surface/volume ratio.^18,30^ A potential approach for WZ phase growth beyond the critical length is the use of a Ga-pulse during growth.^18,25^ A Ga-pulse is executed by momentarily halting the As supply, leading to an accumulation of Ga atoms within the catalyst particle, thereby resetting the Ga/As ratio to a value that enables growth of WZ. However, achieving phase control via multiple Ga-pulses is challenging and requires precise timing for each Ga pulse duration.^25^

**Figure 1:**
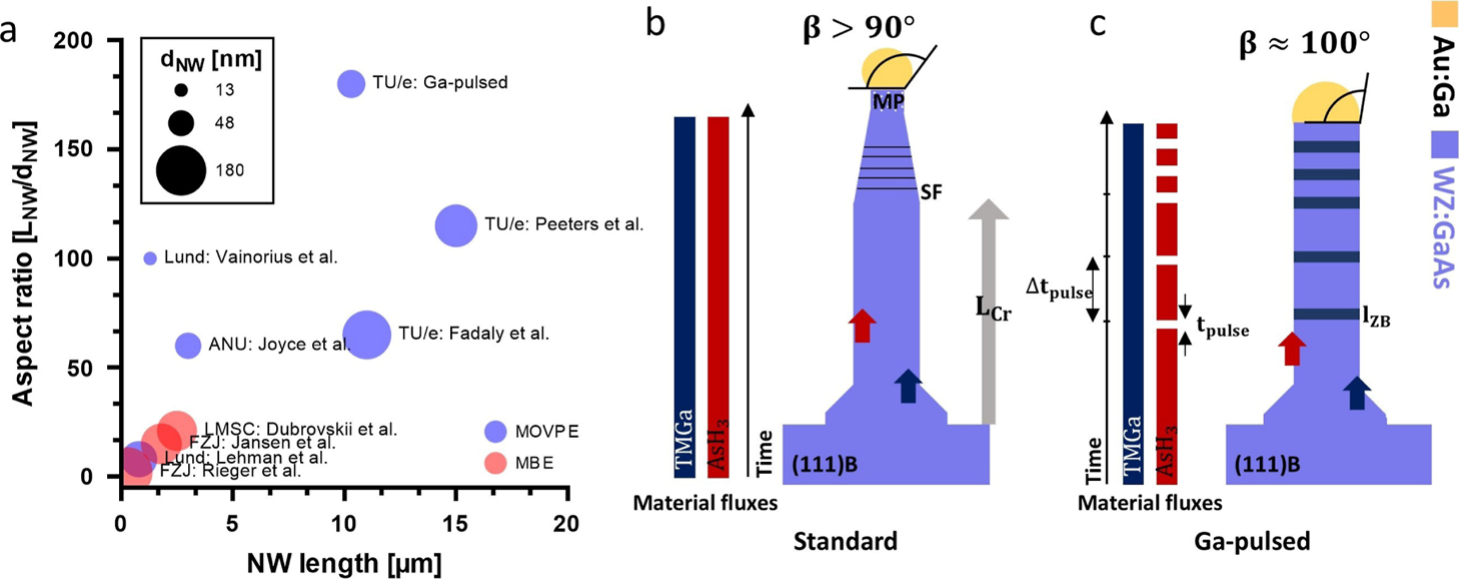
(a) Literature overview of wurtzite GaAs nanowire growth. Aspect ratio (*L*_NW_/*d*_NW_) plotted against NW length. Data points are differentiated by group, growth technique (MOVPE, MBE) and NW diameter which are indicated by text, color and circle size.,^8,10,11,18,22–25,31^ Schematic comparison of standard and Ga-pulsed GaAs NW growth modes. (b) Standard GaAs NW growth scheme with constant material fluxes and critical length. Above critical length, the NW diameter is decreased and the onset of polytypism occurs. (c) In the Ga-pulsed GaAs NW growth, the AsH_3_ flow is continuously stopped, which leads to an accumulation of Ga atoms into the catalyst particle inducing a ZB inclusion.

Here, we report on the crystal phase control of GaAs NWs down to the monolayer regime by Ga-pulsing enabling the growth of high aspect ratio NWs. We systematically analyze the accumulation of Ga atoms within the catalyst particle as a function of pulse time, frequency and position along the NW. Thereby, we controllably increase the contact angle of the catalyst particle inducing the transition from the WZ phase to the zinc blende (ZB), and then back to the WZ phase. This approach generates thin ZB inclusions that serve as markers during growth, which are utilized to fine-tune the pulse duration and frequency to stabilize the WZ phase above the critical length. With this study, we showed the growth of high aspect ratio WZ GaAs NWs up to nearly 200, expanding the platform capabilities of GaAs NWs for the next generation of core/shell heterostructures.

## Experimental Details

GaAs NWs are grown from Au catalysts particles on GaAs (111)B substrates using a low pressure (50 mbar) close coupled showerhead metal organic vapor phase epitaxy (MOVPE) with conditions optimized for the formation of WZ phase. The diameter of the investigated NWs is 57 ± 3 nm measured below the catalyst particle by transmission electron microscopy (TEM). A more detailed description of the standard WZ NW growth process and the sample preparation are depicted in previous studies.^18,25^ Two growth modes are distinguished in this work: the standard growth scheme as well as the Ga-pulsed growth (Figure 1b,c). Both growth modes are facilitated under a V/III ratio of 2.4 and at a growth temperature of 615 °C measured by the thermocouple element. After the growth, the NWs are cooled down rapidly under H_2_ atmosphere too freeze out the Au catalyst particle. The sole addition in the Ga-pulsed growth mode compared to the standard growth is the periodic closure of the AsH_3_ flux for *t*_pulse_ = 2–10 s every Δ*t*_pulse_ = 2.5–5 min. A more detailed description of the growth modes follows.

Figure 1a compares the Ga-pulsed growth of WZ GaAs NWs in this study with selected literature reports from the past two decades. The aspect ratio, defined as NW length divided by diameter, is plotted as a function of NW length. While not exhaustive, the data set highlights representative examples of WZ GaAs NW growth. We distinguish between MOVPE and MBE growth, whereas the highest aspect ratios have been achieved by MOVPE. Unit now, long WZ GaAs NW with a length longer than 10 μm, were only achieved for diameters exceeding 130 nm limiting the aspect ratio to around 100. All reference MOVPE studies are grown by the standard growth scheme, in which continuous precursor flows are utilized. In contrast, our study uses a Ga-pulsed scheme, marking the first reported growth of WZ GaAs NWs reaching an aspect ratios well above 100.

In this work, we utilize Ga-pulses (Figure 1c) to overcome the WZ growth limit of the standard growth scheme (Figure 1b), which has been reported by refs 18 and 25 . In the standard growth scheme, NWs are grown under a continuous flow of trimethylgallium (TMGa) and arsine (AsH_3_) precursors with a V/III ratio of 2.4. By this, the NWs are grown in the WZ phase up to the critical length *L*_CR_. At the critical length, the contact angle of the NWs is decreased below the WZ growth window (<90°) and the crystal phase switches. The diameter of the NW reduces and the onset of polytypism is observed. The crystal phase changes from WZ to mixed phase (MP), which is defined as a mixture of ZB and WZ phase. The critical length varies as a function of the NW diameter and limits the aspect ratio of thin GaAs NWs to below 100. To increase the WZ GaAs aspect ratio, we introduce the utilization of Ga-pulses during the growth as depicted in Figure 1c. A Ga-pulse is defined as the interruption of the AsH_3_ precursor flow for a certain time slot, *t*_pulse_, while the flux of TMGa is not altered. We intend to use the excess Ga species in the gas phase to the accumulation of Ga atoms in the catalyst particle which increase the contact angle into the ZB growth window resulting in a ZB inclusion with thicknesses of *I*_ZB_. Afterward, the AsH_3_ precursor gases are reintroduced into the reactor with a V/III ratio of 2.4, which should restore the WZ phase. To stabilize the crystal phase for longer growth segments, Ga-pulses are introduced periodically with a period of Δ*t*_pulse_.

To increase the WZ GaAs aspect ratio, we introduce the utilization of Ga-pulses during the growth as depicted in Figure 1c. A Ga-pulse is defined as the interruption of the AsH_3_ precursor flow for a certain time slot, *t*_pulse_, while the flux of TMGa is not altered. We intend to use the excess Ga species in the gas phase to the accumulation of Ga atoms in the catalyst particle which increase the contact angle into the ZB growth window resulting in a ZB inclusion with thicknesses of *I*_ZB_. Afterward, the AsH_3_ precursor gases are reintroduced into the reactor with a V/III ratio of 2.4, which should restore the WZ phase. To stabilize the crystal phase for longer growth segments, Ga-pulses are introduced periodically with a period of Δ*t*_pulse_.

It is worth noting that a gradual change of the V/III ratio was experimentally tested and did not lead to prolonged WZ phase growth (see Figure S14 in the Supporting Information). We ascribe this to the spread of the critical length within an array and the subsequent challenge to precisely time the V/III ratio change.

## Results and Discussion

The effect of a Ga-pulse on the contact angle and the crystal phase of WZ GaAs NWs is investigated by TEM. Therefore, WZ GaAs NWs are grown with a diameter of 57 ± 3 nm and a length of approximately 1.0 ± 0.1 μm (*L*_NW_ < *L*_CR_). We study the effect of a Ga-pulse (*t*_pulse_ = 10 s) by preparing five samples. One sample has been grown for 30 min using WZ growth parameters. The remaining four samples receive a Ga-pulse after ∼30 min of WZ growth and are then grown further under WZ conditions for different times: 0, 5, 10, and 20 s. For each sample, the contact angle and crystal phase in the top segment of 4−6 NWs have been analyzed ex-situ using high-resolution scanning TEM.

An exemplary high angle annular dark field (HAADF) STEM micrograph of a NW grown for 20 s after the Ga-pulse is depicted in Figure 2a. The contact angle between catalyst and the NW, β, and the crystal phase underneath the catalyst particle can be seen to be close to 90° for this specific wire. Directly underneath the particle, we observe the WZ crystal phase with a small ZB inclusion, *l*_ZB_, marked in yellow. The ZB inclusion is defined by the characteristic ABC stacking, which can also inherit a twinning event. To simplify the analysis, the ZB inclusion thickness is defined as a ZB segment with less than 5 monolayer (ML) WZ in-between.

**Figure 2:**
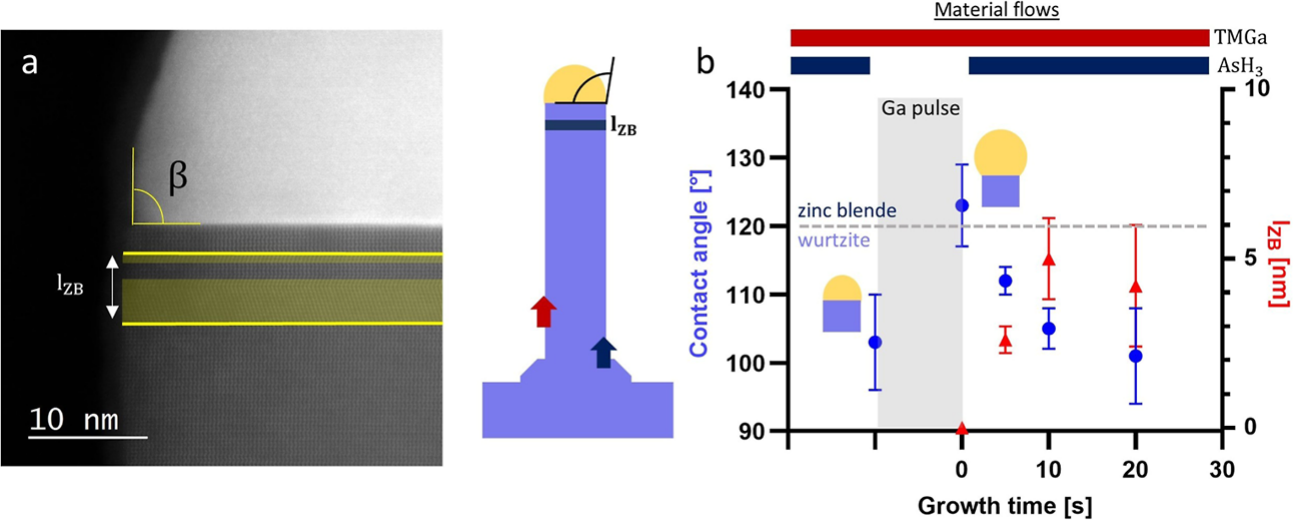
Contact angle evolution and crystal phase switch induced by Ga-pulse. (a) HR-TEM image of the crystal phase directly underneath the Ga–Au catalyst particle. The ZB inclusion is highlighted in yellow. The NW growth is continued for 10 s after the Ga-pulse. (b) The contact angle and ZB inclusion thickness as a function of the growth time after a 10 s Ga-pulse are shown.

The occurrence of the ZB inclusions can be explained by the momentarily increase of the contact angle by the Ga-pulse into the ZB growth regime (>120°)^20^ observed in Figure 2b. The contact angle of the investigated NWs as well as the ZB inclusion thickness as a function of the growth time are shown in Figure 2b. The TEM measurements are performed ex-situ. While ex-situ contact angles may vary from in situ values, we expect that consistent measurement conditions yield results from which a trend can be extracted. The contact angle is raised from 103 ± 7° to 123 ± 6° after the *t*_pulse_ = 10 s Ga-pulse, which is attributed to the accumulation of Ga-atoms in the catalyst particle. With the reintroduction of AsH_3_ into the reactor, the excess Ga atoms are consumed, and the contact angle decreases back to 101 ± 6° within 20 s of growth time. By examining the crystal phase evolution beneath the catalyst particle, we observe that the ZB inclusion grows to a thickness of 4–5 nm, occurring within the first 10 s after the Ga-pulse. A ZB segment is grown for a duration *t*_ZB_ < 10 s. Aftwards, the crystal phase transitions back to the WZ phase. It is thus possible to alter the contact angle by Ga-pulsing, which enables a continuous stabilization of the contact angle above previous limits such as the critical length. The ZB inclusions can be used to probe the contact angle during growth.

A more precise control of the crystal phase can be obtained by altering the Ga-pulse time *t*_pulse_. Here, we show a systematic analysis of the ZB inclusion thickness for different pulse durations *t*_pulse_ from 2 to 10 s. As in the previous study, the nanowire length remains below the critical length (*L*_NW_ < *L*_CR_). After the Ga-pulse segments, the NW growth is continued for 10–20 s with the standard growth parameters. The crystal phase of the NW top segments are shown in Figure 3a–d. The crystal phase underneath the catalyst is predominantly WZ. ZB segments introduced by the Ga-pulse are highlighted in yellow.

**Figure 3:**
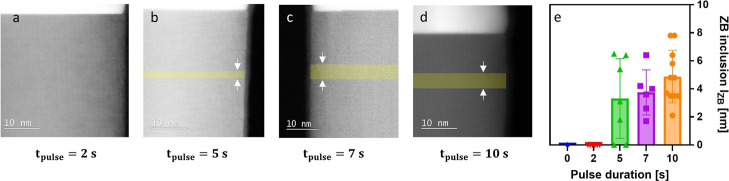
Crystal phase evolution as a function of pulse duration. Atomic resolution HAADF-STEM images depicting the crystal phase underneath the catalyst particle with different pulse durations of (a) 2 s, (b) 5 s, (c) 7 s and (d) 10 s. The growth continues for 20 s after the Ga-pulse. (e) ZB inclusion length vs pulse duration.

For the shortest pulse time *t*_pulse_ = 2 s, there is no small ZB segment detectable underneath the catalyst particle. This implies that not enough additional Ga could be supplied by the Ga-pulse to increase the contact angle of the catalyst particle into the ZB growth regime β > 120.^20^

For *t*_pulse_ ≥ 2 *s*, the ZB inclusion length increases as a function of the pulse duration. This hints on the possibility of precisely tuning the catalyst particle dimensions, particularly the contact angle, by Ga pulsing. The ZB inclusion length as a function of the Ga-pulse time is depicted in Figure 3e. The ZB inclusion length increases from 0 to ∼6 nm for a 10 s Ga-pulse. The uncertainty of the ZB inclusion length is explained by two factors. First, the NW-to-NW variations of the initial contact angle, as indicated by the standard deviation (see Figure 2b). Second, the hysteresis effect observed for the switching between the ZB and WZ growth regimes.^20,21^

For *t*_pulse_ ≥ 7 *s*, we observe vapor–solid (VS) GaAs shell growth around the ZB inclusions. A detailed description of this shell growth is given in the Supporting Information. To maintain high template quality for heteroepitaxy, untapered NWs are preferred. To grow untapered NWs, *t*_pulse_ = 4–5 are utilized for the upcoming Ga-pulse evaluation along the NW length.

Next, the effect of Ga-pulsing as a function of position along the NW length is investigated. Therefore, multiple Ga-pulses are executed during NW growth separated by time intervals Δ*t*_pulse_. Three individual NWs are investigated. The initial 30 min of NW growth are performed under standard WZ growth parameters analogous to the studies discussed above. Following this, Ga-pulses with a duration of *t*_pulse_ = 5 s are conducted separated by intervals of Δ*t*_pulse_ = 2.5–5 min to introduce short ZB segments. The NWs are in total grown for 1.5 h (with standard WZ growth parameters) to a length of *L*_NW_ = 1.7 ± 0.2 μm, which is slightly below the critical length of 2.5 ± 0.5 μm.

Subsequently, the ZB inclusion thickness induced by the Ga-pulse as well as the distance between the ZB inclusions are studied by TEM. The latter is referred to as WZ segments. The detailed growth and analysis schemes are shown in SI Figures S1 and S6.

In Figure 4a, a TEM micrograph of a pulsed grown WZ GaAs NW is shown. The ZB inclusion as well as stacking faults (SFs) in the WZ phase can be observed as contrast lines. The SFs are caused by instabilities of the crystal phase and are defined by a thickness of ≤3 ML. The material flows and the corresponding phase changes, ZB inclusions, are highlighted. Overall, 15 Ga-pulses are conducted for this NW *t*_pulse_(*L*_NW_). The ZB inclusion length is investigated as a function of the NW position (distance from the catalyst particle). As can be seen in Figure 4b, the ZB inclusion thickness varies between 1–9 nm. The ZB inclusions’ length, *d*_ZB_, decreases toward the top of the NW, which is highlighted by the fit line.

**Figure 4:**
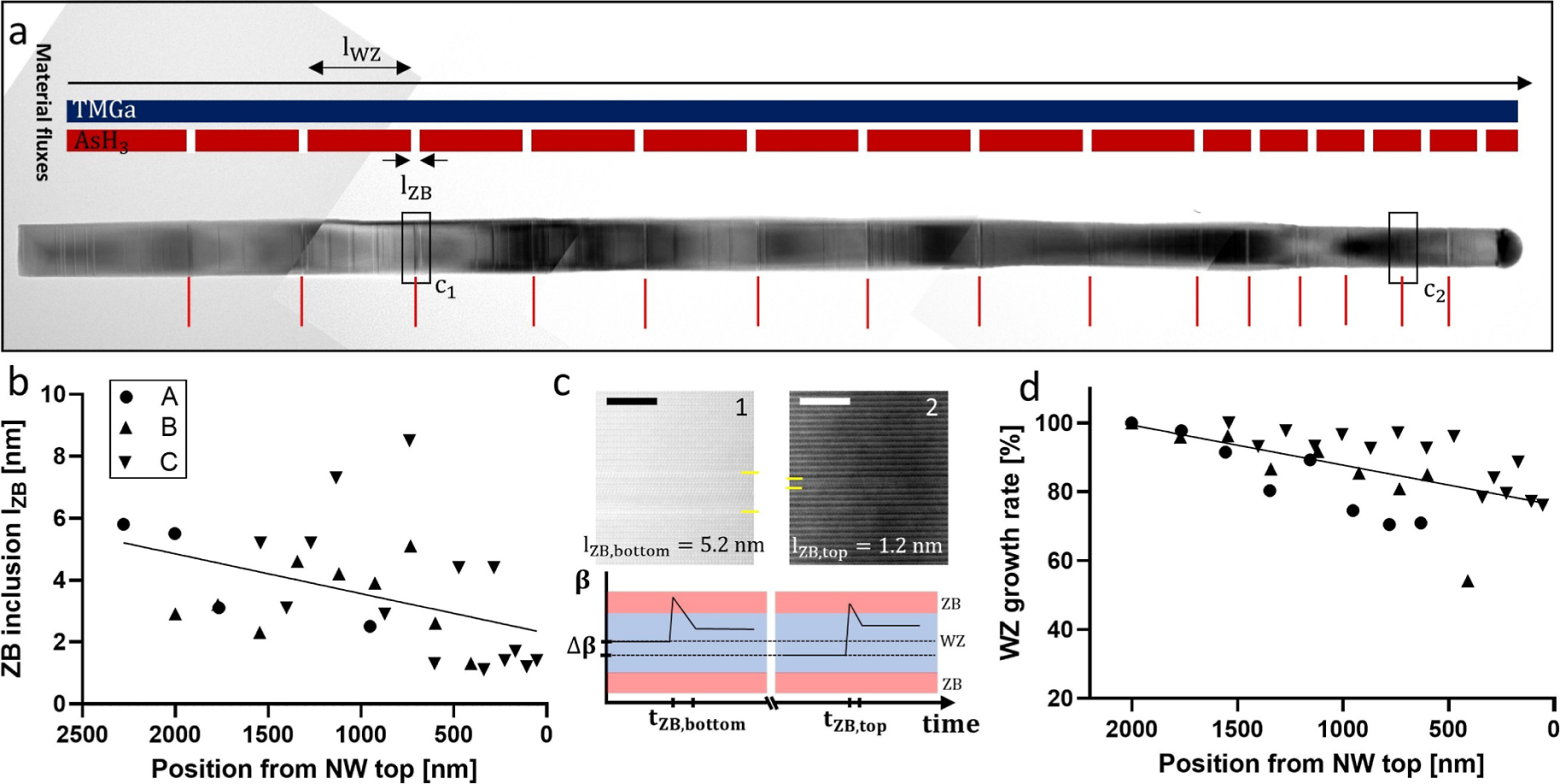
Variation in WZ GaAs NW growth examined by Ga-pulsing. (a) TEM micrograph of the pulsed GaAs NW C. The materials fluxes and Ga-pulse positions are depicted highlighting the ZB inclusions. The interval between the Ga-pulses varies from bottom (Δ*t* = 5 min) to top (Δ*t* = 2.5 min). The scale bar corresponds to 100 nm. (b) ZB inclusion length along the NWs axis. The line represents the linear regression curve with a slope of 0.001. (c) HR-TEM images of ZB inclusions at the bottom and at the top of the NW. The scale bar corresponds to 5 nm. A schematic overview of the expected contact angle evolution is given for the bottom and top, respectively. (d) The WZ growth rate in percentage as a function of the position along the NW. The line represents the linear regression curve with a slope of 0.01.

In Figure 4c, HR-TEM images of ZB inclusion at the bottom and top of the NW are displayed. We attribute the reduced ZB inclusion thickness to a reduced equilibrium contact angle (β_bottom_ > β_top_) of the catalyst particle for a NW length close to the critical length (see schematic in Figure 4c).^25^ We suggest that the change in contact angle (Δβ) influences the local ZB growth time, longer at the bottom than the top, leading to a thicker ZB segment at the bottom. This motivates the need for elongated pulse durations for longer NW growth experiments, *t*_pulse_(*L*). However, the precision of these measurements is likely limited by the thickness fluctuations of the ZB inclusions observed in Figures 2 and 3.

For a more detailed analysis, we examine the WZ growth rate, distance between the ZB inclusions divided by *t*_pulse_, as a function of the NW length. It is worth noting that we approximate Δ*t*_pulse_ ≈ *t*_WZ_ with *t*_ZB_ < 10 s (see Figure 2). To exclude NW-to-NW variations in the growth rate analysis,^18,25^ the growth rate is presented in Figure 4d relative to the initial growth rate of each NW. We observe a growth rate decrease from bottom to the top of the NW by approximately 20%. The growth rate of GaAs NWs depends on three contribution pathways: (a) the direct impingement of precursors on the catalyst particle as well as diffusion from adatoms impinging (b) on the side facets of the NW and (c) on the substrate. The contribution from adatom diffusion via pathways (b) and (c) is expected to vary as a function of the NW length, which changes the effective ratio of As and Ga atoms contributing to the NW growth.^19,24^ However, below the critical length, the variations are expected to be minor, since the WZ phase is stable with low SF density.^25^ The decrease in growth rate cannot be explained by the sole reduction of available surface area of the catalyst particle, which is expected to shrink from around 100° to ∼90° close to the critical length. This shrinkage corresponds to a collection area decrease of 5%.^8,25^ Therefore, we conclude that the growth rate is likely limited not only by the reduction in contact angle but also by a decrease in adatom diffusion; the axial growth rate of the NWs decreases due to a limited supply of Ga and As adatoms to the growth front. A theoretical discussion is beyond the scope of this work.

With the above-mentioned results, we designed a pulse scheme that enables the growth of WZ GaAs NWs significantly longer than the *L*_CR_ of approximately 2.5 μm for 60 nm diameter NWs.^25^ The pulse duration is varied from *t*_pulse_(*L*_NW_) = 4–7 s as a function of the NW length. The pulse intervals are decreased around *L*_CR_ to improve the crystal phase stability with Δ*t*_pulse_(*L*_NW_) = 5 to 2.5 min. The effect of modified pulse intervals is illustrated in Figure S8 in the Supporting Information. The precise pulse scheme is discussed in Figure S1 in the Supporting Information. Further refinements to the pulse scheme aimed at WZ phase control without ZB inclusions may be possible by optimizing the pulse frequency and duration. However, this falls outside the scope of this study. For the growth beyond the original critical length, *L*_CR_, an optimum pulse duration of 7 s is experimentally determined (see Supporting Information). Overall, 122 Ga-pulses are utilized, and the GaAs NWs are grown for 4.5 h. The crystal phase evolution of 8 individual NWs grown with the pulse scheme is examined and compared to the crystal phase evolution of standard grown NWs for the same growth time.

The morphology of the long GaAs NW grown with constant flows is shown in Figure 5a. The NWs are grown significantly longer than the critical length, while only minor tapering is detectable (see *L*_Cr_ in Figure 5a). The crystal phase is mixed WZ/ZB phase at the top of all investigated NWs (see Figure 5b), which is in line with previous studies.^25^

**Figure 5:**
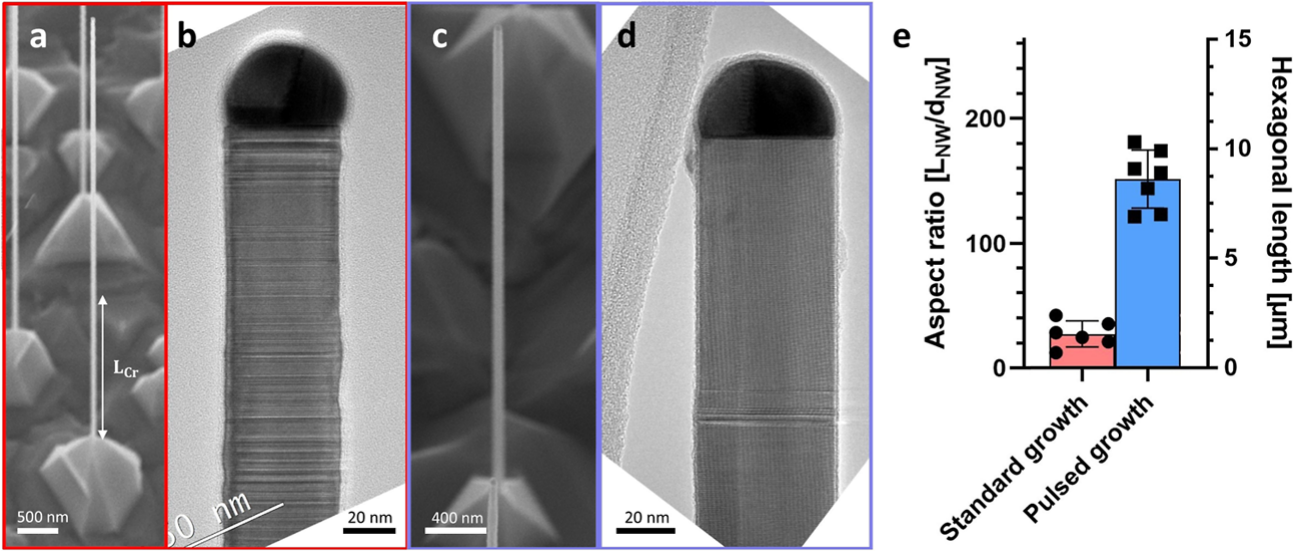
GaAs MW growth beyond critical length. (a) SEM image of a GaAs NW grown for 4.5 h with the standard recipe using constant material flows. (b) TEM image of the NW top highlighting the mixed-phase crystal structure underneath the catalyst particle. (c) SEM image of a Ga-pulsed GaAs NW grown for 4.5 h. (d) The WZ crystal phase under a Ga-pulsed NW is verified by TEM. (e) The aspect ratio and hexagonal length in GaAs NWs from the standard and pulsed growth schemes are compared. The SEM images are taken under an angle of 30°.

The pulsed GaAs NW is depicted in Figure 5c. The NW morphology is comparable to the unpulsed NWs, while we observed VS shell growth around large ZB inclusions for some NWs at the bottom, which results in an on average slighty tapered NW morphology (∼0.02). A detailed overview is given in the Supportin Information. The crystal phase at the top of the NWs is shown in Figure 5d. Here, in contrast to the standard growth, we identify the WZ crystal phase along the NW. The WZ lengths of both growth schemes are compared in Figure 5e. In the standard growth process, the WZ segment reaches a length of 1.6 ± 0.5 μm before transitioning to mixed phase. In contrast, our pulsed growth scheme enabled a significant increase in WZ segment length to 9 ± 1 μm, with ∼10 SF/μm and ZB inclusions induced by Ga pulses. This represents an average aspect ratio increase from ∼30 without pulses to ∼150 with pulses, which proves the functionality of the Ga-pulsing approach.

## Conclusion and Outlook

In conclusion, we investigated the effect of Ga-pulsing on WZ GaAs NW growth and developed a pulsing scheme to stabilize the crystal phase beyond the current limitations. The phase tuning characteristics of Ga-pulses are identified based on variations in pulse duration, frequency, and position along the NW length. The critical length, which was previously the upper limit of the WZ phase, was significantly extended by using Ga-pulses to stabilize the growth of WZ GaAs NW, achieving aspect ratios of nearly 200. This introduced pulse method gives important insights in the growth dynamics and particularly on the depletion of precurors from the catalyst. This can be used to develop more advanced growth schemes to yield even longer phase pure wires, or crystal phase superlattices. In addition, this study can be extended to various material systems and enables an unprecedentend level of crystal phase control in semiconductor nanowires.

## Supplementary Material



## Notes

A detailed discussion of the Ga-pulsed growth scheme, Ga-pulsed growth approaching the critical length, the ZB segment measuring procedure, vapor–solid growth around ZB inclusions, the crystal phase analysis as well as Ga-pulse as a function of diameter are presented in the Supporting Information. Raw data available in DOI: 10.5281/zenodo.15862209 (PDF)

## References

[1] Barrigón E., Heurlin M., Bi Z., Monemar B., Samuelson L. (2019). Synthesis
and Applications of III-V Nanowires. Chem. Rev..

[2] Zhang Y., Wu J., Aagesen M., Liu H. (2015). III–V nanowires and nanowire
optoelectronic devices. J. Phys. D Appl. Phys..

[3] Balaghi L., Bussone G., Grifone R., Hübner R., Grenzer J., Ghorbani-Asl M., Krasheninnikov A. V., Schneider H., Helm M., Dimakis E. (2019). Widely tunable
GaAs
bandgap via strain engineering in core/shell nanowires with large
lattice mismatch. Nat. Commun..

[4] Glas F., Harmand J. C., Patriarche G. (2007). Why does wurtzite
form in nanowires
of III-V zinc blende semiconductors?. Phys.
Rev. Lett..

[5] Joyce H. J., Gao Q., Tan H. H., Jagadish C., Kim Y., Fickenscher M. A., Perera S., Hoang T. B., Smith L. M., Jackson H. E. (2008). High Purity GaAs Nanowires Free of Planar Defects: Growth and Characterization
**. Adv. Funct. Mater..

[6] Dick K. A. (2010). Control of III–V nanowire crystal structure by growth parameter
tuning. Semicond. Sci. Technol..

[7] Krogstrup P. (2010). Structural phase control in self-catalyzed
growth of GaAs nanowires
on silicon (111). Nano Lett..

[8] Rieger T., Lepsa M. I., Schapers T., Grützmacher D. (2013). Controlled
wurtzite inclusions in self-catalyzed zinc blende III-V semiconductor
nanowires. J. Cryst. Growth.

[9] Hauge H. I. T. (2015). Hexagonal Silicon Realized. Nano
Lett..

[10] Vainorius N. (2016). Wurtzite GaAs Quantum
Wires: One-Dimensional Subband Formation. Nano
Lett..

[11] Fadaly E. M. T. (2020). Direct-bandgap emission
from hexagonal Ge and SiGe
alloys. Nature.

[12] Peeters W. H. J., van Lange V. T., Belabbes A., van Hemert M. C., Jansen M. M., Farina R., van Tilburg M. A. J., Verheijen M. A., Botti S., Bechstedt F. (2024). Direct bandgap quantum wells in hexagonal Silicon Germanium. Nat. Commun..

[13] McIntyre P. C., Fontcubertai Morral A. (2020). Semiconductor
Nanowires: To Grow or Not to Grow?. Mater. Today
Nano.

[14] Hauge H. I. T., Conesa-Boj S., Verheijen M. A., Koelling S., Bakkers E. P. A. M. (2017). Single-Crystalline
Hexagonal Silicon-Germanium. Nano Lett..

[15] Zhou Z., Ou X., Fang Y., Alkhazraji E., Xu R., Wan Y., Bowers J. E. (2023). Prospects
and applications of on-chip lasers. eLight.

[16] van
Lange V. T., Dijkstra A., Fadaly E. M. T., Peeters W. H. J., van Tilburg M. A. J., Bakkers E. P. A. M., Bechstedt F., Finley J. J., Haverkort J. E. M. (2024). Nanosecond
Carrier Lifetime of Hexagonal Ge. ACS Photonics.

[17] van
Tilburg M. A. J., Farina R., van Lange V. T., Peeters W. H. J., Meder S., Jansen M. M., Verheijen M. A., Vettori M., Finley J. J., Bakkers E. P. A. M. (2024). Stimulated emission from hexagonal silicon-germanium nanowires. Commun Phys.

[18] Fadaly, E. M. T. Epitaxy of Hexagonal SiGe Alloys for Light Emission, Ph.D. Thesis, Eindhoven University of technology, The Nethelrands 2021.

[19] Dubrovskii V. G., Sibirev N. V., Harmand J. C., Glas F. (2008). Growth kinetics and
crystal structure of semiconductor nanowires. Phys. Rev. B.

[20] Jacobsson D. (2016). Interface dynamics and
crystal phase switching in GaAs nanowires. Nature.

[21] Panciera F. (2020). Phase Selection in Self-catalyzed GaAs Nanowires. Nano Lett..

[22] Joyce H. J., Wong-Leung J., Gao Q., Tan H. H., Jagadish C. (2010). Phase perfection
in zinc blende and wurtzite III- V nanowires using basic growth parameters. Nano Lett..

[23] Lehmann S., Jacobsson D., Dick K. A. (2015). Crystal phase control in GaAs nanowires:
Opposing trends in the Ga- and As-limited growth regimes. Nanotechnology.

[24] Jansen M. M. (2020). Phase-Pure Wurtzite
GaAs Nanowires Grown by Self-Catalyzed Selective
Area Molecular Beam Epitaxy for Advanced Laser Devices and Quantum
Disks. ACS Appl. Nano Mater..

[25] Peeters W. H. J., Vettori M., Fadaly E. M. T., Danescu A., Mao C., Verheijen M. A., Bakkers E. P. A. M. (2024). Onset of uncontrolled polytypism
during the Au-catalyzed growth of wurtzite GaAs nanowires. Phys. Rev. Mater..

[26] Ramdani M. R., Harmand J. C., Glas F., Patriarche G., Travers L. (2013). Arsenic pathways in self-catalyzed
growth of GaAs nanowires. Cryst. Growth Des..

[27] Pishchagin A. (2021). Dynamics of Droplet Consumption in Vapor-Liquid-Solid III-V Nanowire
Growth. Cryst. Growth Des..

[28] Rudolph D. (2011). Direct observation of a noncatalytic growth regime for GaAs nanowires. Nano Lett..

[29] Tambe M. J., Ren S., Gradečak S. (2010). Effects of
gold diffusion on n-type
doping of GaAs nanowires. Nano Lett..

[30] Li M., Mayer T. S., Sioss J. A., Keating C. D., Bhiladvala R. B. (2007). Template-grown
metal nanowires as resonators: Performance and characterization of
dissipative and elastic properties. Nano Lett..

[31] Dubrovskii V. G., Kim W., Piazza V., Güniat L., Fontcuberta i Morral A. (2021). Simultaneous
Selective Area Growth of Wurtzite and Zincblende Self-Catalyzed GaAs
Nanowires on Silicon. Nano Lett..

