# Supplementary material for: Growth of High Aspect Ratio Wurtzite GaAs Nanowires
Source: Cryst Growth Des. 2025 Aug 22;25(17):7105–11. doi: 10.1021/acs.cgd.5c00312 (PMC12412090; doi:10.1021/acs.cgd.5c00312)
Supplement: Supplementary file 1 [file cg5c00312_si_001.pdf]

Supplementary Information: Growth of high aspect ratio wurtzite GaAs nanowires

M.M. Jansen,[a] **W.H.J. Peeters**,[a] **D. Lamon**,[a] **M.F. Schouten**,[a] **M.A. Verheijen**,[a,b] and **E.P.A.M. Bakkers**[a]

[a] *Department of Applied Physics, Eindhoven University of Technology, Groene Loper 19, 5612AP Eindhoven, The Netherland*
[b] *Eurofins Materials Science BV, High Tech Campus 11, 5656 AE Eindhoven, The Netherlands*

Corresponding Author
Marvin Marco Jansen * E-Mail: m.m.jansen@tue.nl, Erik Bakkers * E-Mail: e.p.a.m.bakkers@tue.nl

1. Ga-pulsed growth scheme

The GaAs NWs presented in this work are grown on GaAs (111)B substrates in a low-pressure Aixtron close coupled showerhead MOVPE reactor. A detailed overview of the pre-growth fabrication steps as well as the WZ GaAs NW growth are given in previous work.[1] In short, the native oxide of GaAs (111)B substrates is removed by diluted NH_4OH. PMMA-950K-A2 resist is spin-coated, and hole patterns are created using electron beam lithography. After resist development, a 6 nm layer of gold is deposited and the PMMA/Au layer stack is lifted off by PRS3000, acetone, and isopropanol. Finally, resist residuals are removed by oxygen plasma and, after a final NH_4OH step the samples are introduced into the reactor for growth.

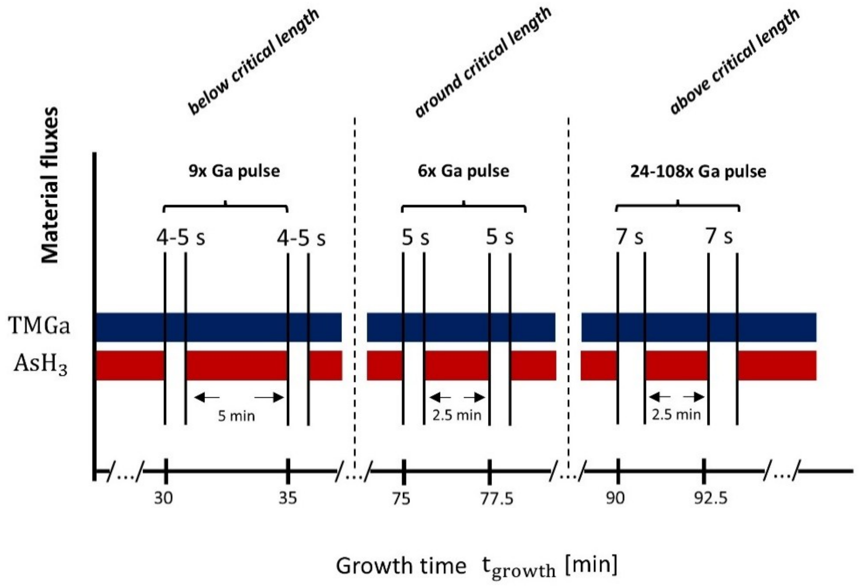

Figure S1: Growth scheme for Ga-pulsed NW growth above the critical length. The material fluxes are depicted as a function of time for three distinct pulsing sections: (1) below the critical length, (2) around critical length and (3) above critical length. The pulse duration and periodicity varies in these sections.

In the growth reactor, the GaAs samples are annealed at 635 °C (set by the thermocouple element) at high AsH3 flow to clean the surface and establish an Au-Ga eutectic. Next, the growth conditions are established with a V/III flow ratio of 2.4 with an AsH3 molar flow of 0.373 sccm and a TMGa flow of 0.157 sccm at a growth temperature of 615 °C. The GaAs NWs are grown for 30 minutes under these conditions. Next, the Ga-pulse scheme is conducted. The scheme is divided into three growth regimes: below, around and above the critical length. First, the pulse regime "below the critical length", depicted in Figure S1, is utilized. The Ga-pulse

duration is set to $\text{t}_{\text{pulse}} = 4 - 5\,\text{s}$ every $\Delta\text{t}_{\text{pulse}} = 5\,\text{min}$ to monitor the NW growth. Next, the pulse frequency is increased to $1/\Delta\text{t}_{\text{pulse}} = 1/2.5\,\text{min}^{-1}$ to stabilize the contact angle in the pulse regime "around the critical length". It is worth noting that the NWs examined in Figure 4 of the main text are grown with the first two pulse regimes. Thereby, one NW is grown with the depicted scheme, while two NWs are grown with only $\Delta\text{t}_{\text{pulse}} = 5\,\text{min}$ around the critical length for the same total growth time, which results in 12 pulses for 12 NWs and 15 pulses for the other. Finally, the Ga-pulse duration is increased to 7 s in the growth regime identified as "above critical length". The full pulse scheme is utilized for the pulsed NWs shown in Figure 5 of the main text.

A. Ga-pulsed GaAs NW growth for 2.5 hr

After the GaAs NW growth for 1.5 hours, as shown in Figure 4 of the manuscript, the growth time is extended by an additional hour. The first GaAs NW growth above the critical length is conducted with a growth time of 2.5 hr. The Ga-pulse scheme shown in Figure S1 is utilized with all three distinct pulsing sequences. For the final sequence, named "above critical length", 24 Ga-pulses are conducted. An SEM micrograph of the GaAs NWs is shown in Figure S2a. The NWs achieve a length of $3.3 \pm 0.4\ \mu\text{m}$ with nearly no tapering, which is above the critical length of approx. 2.5 µm. The crystal phase purity is verified by HR-TEM along the NW. An exemplary NW is shown in Figure S2b-c. We verify the WZ crystal phase at the top. The ZB inclusions induced by the Ga-pulses are marked by black lines in Figure S2c.

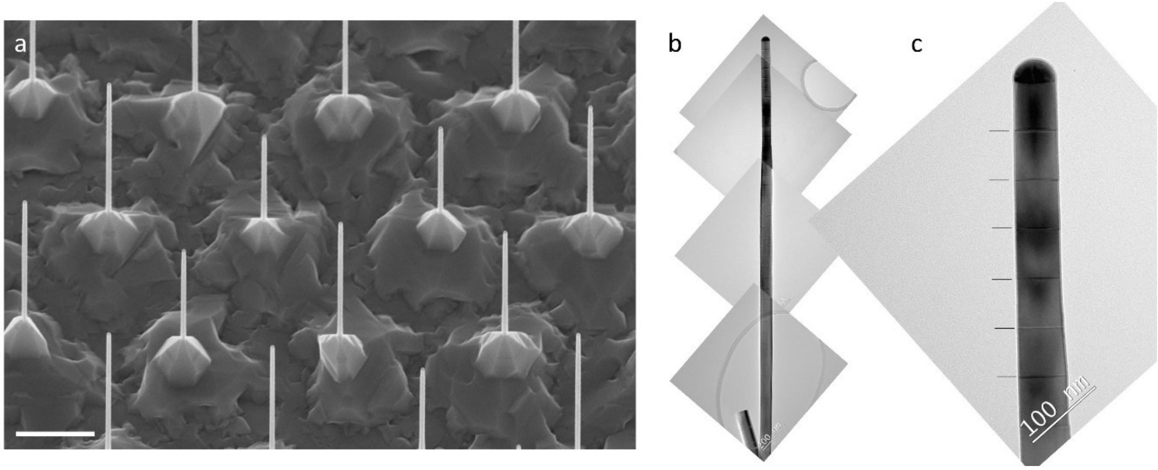

Figure S2: Pulsed WZ GaAs NW growth around critical length. (a) SEM micrograph of NW array grown for 2.5 hr with the pulsed growth scheme introduced in Fig.S1. The scale bar is 1 µm. (b) TEM analysis of crystal phase of a 2.5 hr grown NWs. (c) Zoom-in of the crystal phase at the top. Verifying WZ phase and highlighting the induced ZB inclusions as contrast lines.

B. GaAs NW growth for 4.5 hr without and with Ga-pulsing

We grew two distinct GaAs NW samples for 4.5 hr – one without and one with Ga-pulsing. SEM overviews of the grown NWs are given in Figure S3a and b. The unpulsed GaAs NWs are depicted in Figure S3a. The morphology of these NWs is nearly untapered and straight with a length of $12 \pm 2\ \mu m$.

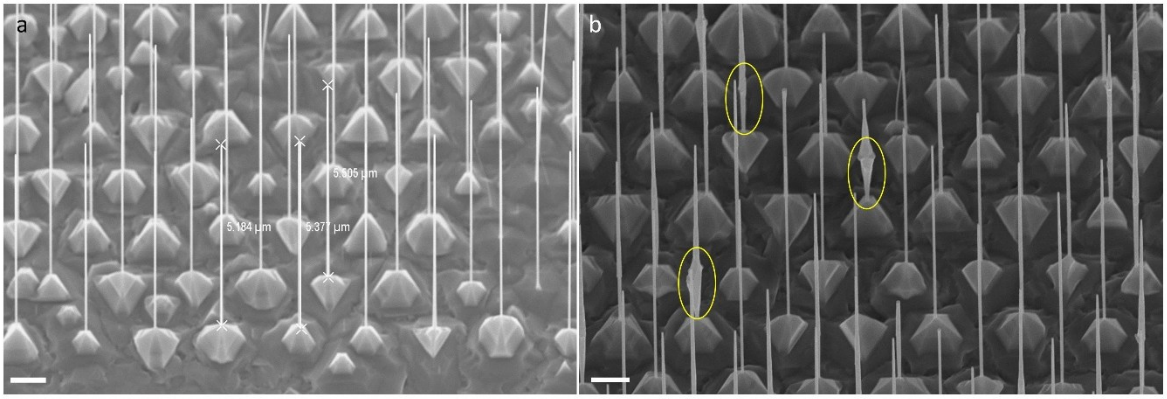

Figure S3: Comparison: Standard NW growth vs pulsed WZ GaAs NW growth above critical length. (a) SEM micrograph of NW array grown for 4.5 hr without Ga-pulsing. (b) GaAs NWs grown for 4.5 hr with pulsing scheme depicted in Fig. S1. GaAs vapor solid growth around the NW bottom is highlighted in yellow. Scale bars are 1 µm.

The GaAs NWs grown with the pulse scheme are shown in Figure S3b. The average length of these NWs is $10 \pm 1\ \mu m$. While the majority of GaAs NWs obtain only slight tapering, there are several wires with large diameter increases around the bottom half of the NWs (highlighted in Fig. S3b). The diameter increases are ascribed to vapor solid (VS) growth of GaAs around ZB inclusions with extended length. A more precise study of the VS growth around ZB inclusions is given in section 4.

2. *GaAs shell growth around zinc blende inclusions*

A. Shell growth as a function of ZB inclusion thickness

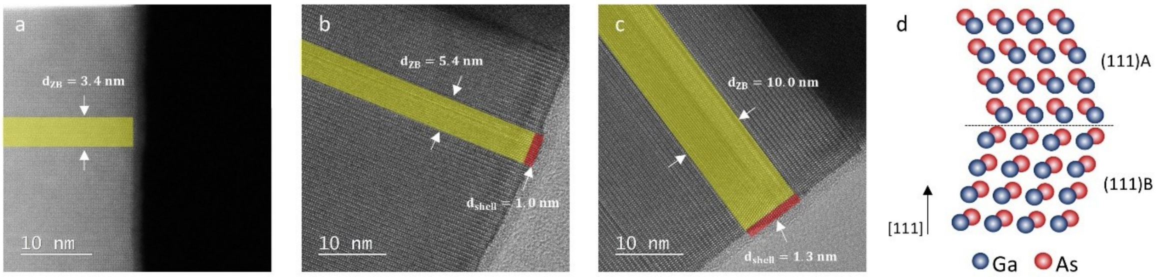

Figure S4: Vapor-solid growth around ZB inclusion as a function of ZB inclusion thickness. TEM images of the crystal phase evolution of the NW top for (a) short, (b) medium and (c) long Ga-pulse durations. ZB inclusions are highlighted in yellow. GaAs shell growth around the ZB segments is depicted in red. (d) Schematic illustration of the facet surface passivation in a twinned ZB inclusion. All images have been acquired along the <11-20> wurtzite / <110> zinc blende zone axis.

Here, the GaAs VS growth around ZB inclusions is investigated as a function of the ZB inclusion length by HR-TEM. Therefore, the crystal phase evolution directly under the catalyst particle is examined for different ZB

inclusion lengths after 20 s of growth. The growth time after the VS growth is kept constant here and will be analyzed in detail in the upcoming chapter. As can be seen in Figure S4a, we do not observe VS growth for ZB inclusions of $\sim$3 nm. However, the onset of VS growth can be observed for ZB thicknesses ≥ 5 nm (see Figure S4b and c). We observe a shell around the ZB inclusion ranging in thickness from 5 – 10 nm of approx. 1 nm. A quantitative study of the shell growth rate as a function of the ZB inclusion thickness cannot be conducted using the current data set.

While the precise mechanism which induces the VS growth around ZB inclusion is unclear, we observe an increased probability of twinning events appearing in larger ZB inclusion. In twinning events, we observe a concave facet shape as well as a change in the surface termination of the sidewall from the As-terminated (111)B to the Ga-terminated (111)A.[2,3] We hypothesize that the concave surface shape can increase the nucleation probability resulting in an enhanced VS growth rate. Furthermore, we speculate that a change in termination can increase the sticking coefficient of Ga or As atoms resulting in the formation of GaAs shells and/or the formation of stacking faults inclined to the growth direction. The latter induces multiple-twinned bulbs at the NW surface (see Figure S5a-d).

B. Shell growth as a function of time

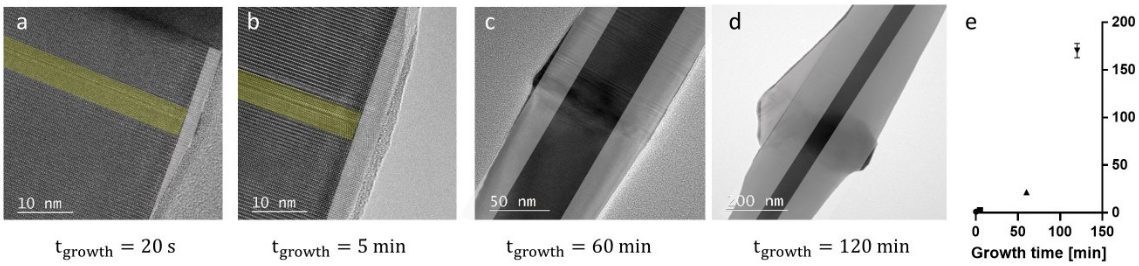

Figure S5: GaAs shell growth around ZB inclusion *as a function of growth time. TEM images highlighting the GaAs shell for growth times of (a) 20 s, (b) 5 min, (c) 60 min and (d) 120 min. (e) GaAs shell thickness as a function of growth time. All images have been acquired along the <11-20> wurtzite / <110> zincblende zone axis.*

Furthermore, we examine the GaAs VS growth as a function of growth time. Here, the pulse duration is set to 10 s, while the growth time after the pulse is varied from 20 s to 120 min. For 20 s of growth, we observe a GaAs shell growth mainly around the ZB inclusion with a thickness of around 1 nm (see Figure S5a). After 5 min of growth (Figure S5b), the GaAs shell thickness is increased to 3 ± 1 nm and we clearly observe shell growth also on the WZ segments. The effect of GaAs shell growth on extended segments of the NW clearly increases further after 60 min of growth time (see Figure S5c). We attribute this to the initial shell growth around the ZB inclusion, which induces layer growth on the side facets of the NW that extends beyond the ZB inclusion. As can be seen in Figure S5d, the VS growth around the ZB inclusion after 120 min significantly increases the NW diameter to above 200 nm compared to pure WZ GaAs NWs with diameter of 50 nm. Furthermore, large, twinned structures are formed around the ZB inclusion. A more detailed analysis of this is shown in Figure S8. The GaAs shell thickness as a function of the growth time is depicted in Figure S5e, highlighting the fast increase in shell formation after 1 – 2 hr. The study was not extended to longer growth times, as this falls outside the scope of the analysis.

It is essential to realize that the shell dimensions are derived from the projections of the wires along the <11-20> wurtzite / <110> zincblende zone axis. In the images of Figure S5, there is a clear contrast between the GaAs core and the GaAs shell. This contrast difference suggests that the shell is thinner in projection than the core. This suggests that on the hexagonally shaped core, rotated facets are grown, i.e {11-20} shell facets on (10-10} core facets.

The VS growth studies motivate the introduction of short Ga-pulses resulting in thin ZB inclusion to examine the NW growth, while not promoting the VS growth of GaAs around ZB inclusions.

3. Measurement procedure

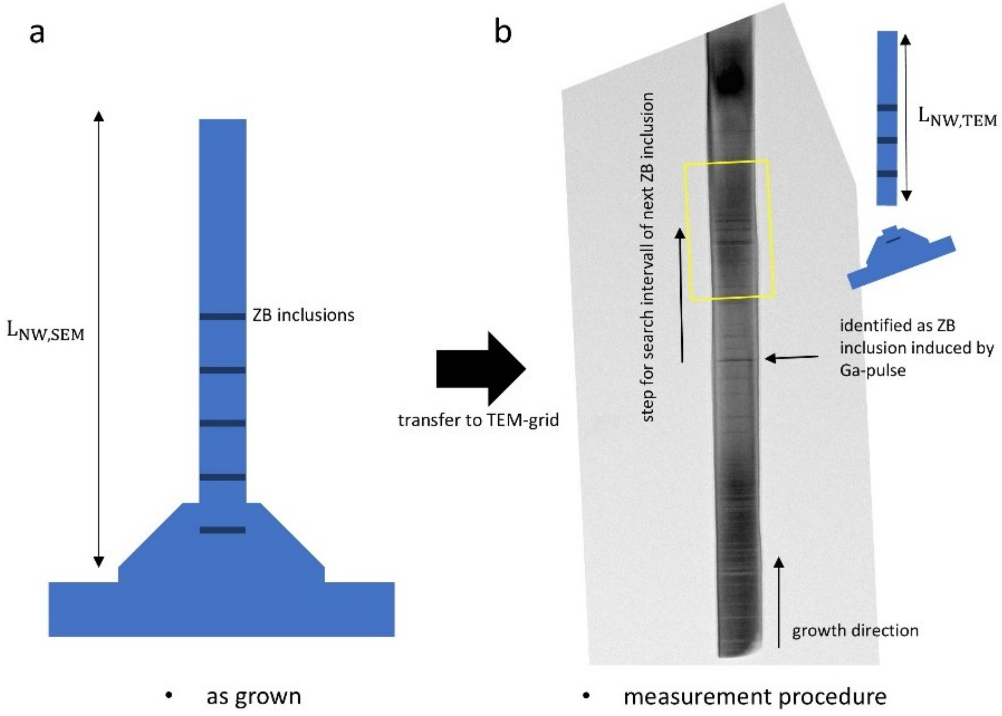

Figure S6: ZB inclusion measurement procedure. (a) NW schematic in as grown geometry. NW and pyramid length are determined in SEM. ZB inclusions can be overgrown by the pyramid. (b) TEM image of the bottom section of a GaAs NW. The initial ZB inclusion identified is marked by a black arrow. Subsequently, the area of the next inclusion is highlighted by the yellow box.

The ZB inclusions are investigated by the following measurement protocol which is followed for all pulsed studies presented in this work. The NW length and pyramid height are measured in the SEM (see Figure S6a). For extended growth times, it is important to point out that ZB inclusions that are induced by Ga-pulses can be overgrown by the pyramid. Using SEM, the average height of the pyramid-like structures at the bottom of the NW as well as the additional average NW length can be examined.[1,2] Taking the dimensions from SEM measurements into account, we can approximate the ZB inclusion positions in GaAs NW after transferring to the TEM grids. An exemplary measurement procedure is depicted in Fig. S6b. We localize the ZB inclusion as the largest ZB inclusion in the close vicinity (± 100 nm) of the expected segment position from the top or, if the SF density at the top is too high, at the bottom.
By this, we can allocate the first detectable pulse. Next, depending on the pulse period Δt_{pulse}, we advance the analysis along the NW and examine a region of 200 ± 100 nm ($t_{pulse} = 5$ min), or 100 ± 50 nm ($t_{pulse} = 2.5$ min). The largest ZB inclusion in the expected area is thereby defined as the next ZB inclusion. This is repeated along the NW.

4. *Crystal phase analysis of a pulsed WZ GaAs NW*

A. SF distribution in WZ segments

First, we observe that applying a Ga-pulse can restore WZ phase growth. Figure S7 shows a TEM image of a GaAs nanowire illustrating this effect. We hypothesize that the Ga pulse increases the contact angle, shifting growth from the mixed-phase (MP) regime back into the WZ regime. This observation supports the hypothesis that a shrinking contact angle underlies the phase transition.

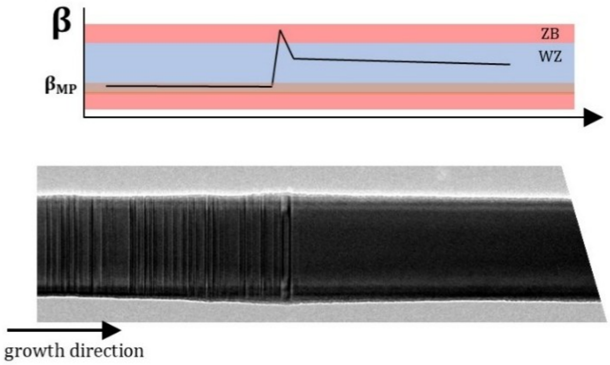

Figure S7: *Hypothesized contact angle evolution after a Ga-pulse. WZ and ZB growth regimes as well as a mixed-phase regime are identified. TEM image of the crystal phase evolution after a Ga-pulse. We identify mixed-phase growth before and WZ growth after the pulse.*

We next investigate how varying the Ga-pulse frequency affects the crystal phase evolution in GaAs nanowires. Specifically, we compare two pulse intervals: 2.5 and 5 minutes. Figure S8a presents a TEM image of a WZ segment with highlighted stacking faults (SFs). Following each pulse, the contact angle returns to the WZ growth regime. However, the exact value within the WZ stability range (100–125°) remains unknown. Since SF density tends to increase during transitions between WZ and ZB phases,[5] we propose that analyzing the number and position of SFs after each pulse may help identify whether the contact angle lies near the boundary (upper or lower) of the WZ growth regime.

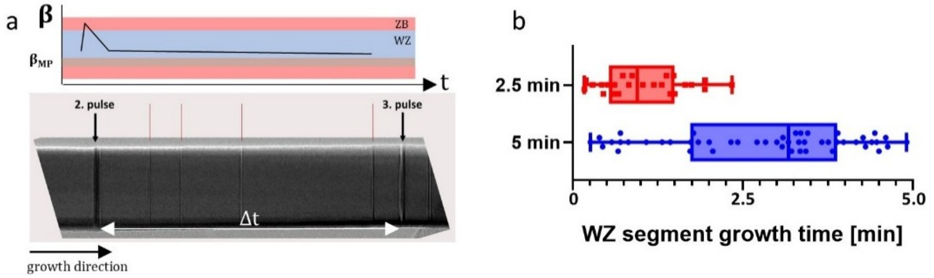

Figure S8: (a) TEM image of the WZ segment between two Ga-pulse-induced ZB inclusions. (b) The SF position as a function of the WZ segment growth time. The SF position for the 2.5 min and 5.0 min intervals are depicted in red and blue, respectively.

Therefore, the SF distribution after the Ga-pulse is examined. This is valuable information to tune the frequency of pulse intervals and enable long WZ GaAs NW growth. The position of SF along the WZ segments from the start to the end of the segments are analyzed. A high density of SFs is associated with a reduction (or increase) of the contact angle towards the ZB growth regime. To compare the SF position for different

segment growth times, the SFs positions are given in relation to the individual segment position. The segments are normalized by the growth times. The diagram showcasing the number of SFs as a function of the relative segment growth time is given in Figure S8b. The line marks the median SFs position, while the boxes extend from the 25th to 75th percentiles.

For 5 min segments, we see an increased number of SFs appearing around 2.5 to 5.0 min. This is explained by a reduction of the contact angle towards the ZB growth regime. To improve the phase stability, 2.5 min segments are introduced. The higher frequency leads to a more uniform SFs distribution over the segment growth time, while most SFs appear < 1 min after the Ga-pulse. We explain these SFs by fluctuations after the Ga-pulse that could be caused by the contact angle being too high. In conclusion, we identify the high frequency as beneficial to control the phase stability. We state that more fine-tuning is needed to stabilize to achieve phase-pure WZ growth.

B. Exemplary sample and measurement overview

In Figure S9, we give an exemplary overview of three WZ GaAs NWs grown with the pulse scheme presented in Figure S1. The crystal phase has been analyzed along the entire NW length by TEM. The crystal phase at the top of each NW is shown in Figure S9b, d and f. The ZB inclusions are highlighted by black lines. In the first NW, we observe mixed-phase at the top (see Figure S8a-b). The other two NWs depicted in Figure S9c-f obtain the WZ phase till the top. One with a higher SF density (c-d) and one with a lower SF density (e-f). We observe a stable WZ phase at the top of 6 out of 8 analyzed NWs.

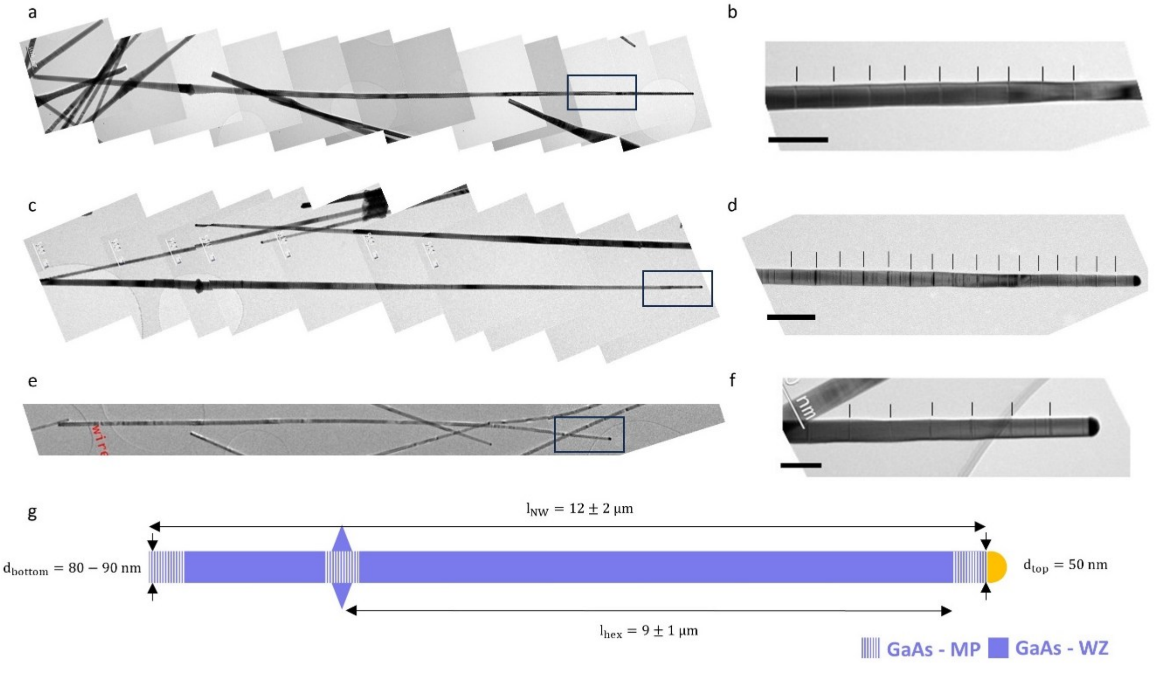

Figure S9: Phase analysis along the entire NW length for three exemplary ones. TEM overview of NWs in (a), (c) and (e). Crystal phase analysis at the top for each NW in (b), (d) and (f). The ZB inclusions are highlighted by black lines. The average NW morphology and crystal phase evolution is depicted in (g).

The contrast difference between the WZ main phase and the ZB inclusions is created by tilting the wire slightly off the <11-20> zone axis. Using this procedure, atomic resolution TEM imaging is not required to identify the positions of the ZB inclusion. For verification of the method, HRTEM images are acquired at selected positions along the NWs. A comprehensive analysis of 8 Ga-pulsed NWs from bottom to top reveals the evolution of their morphology and crystal phase. The study identifies distinct WZ and mixed-phase (MP) regions, with well-

defined hexagonal segments averaging 9 ± 1 µm in length. A schematic overview is depicted in Figure S9g. While some NWs exhibit a consistent wurtzite (WZ) phase throughout (3/8), others display mixed-phase (MP) regions occurring at different points—either at the base (1/8), around the extension growth region (5/8), and/or at the outermost (2/8) top.

Next, we analyze the crystal phase evolution of one exemplary NW. We observe VS shell growth around thick ZB segments at the bottom third of the NWs. The precise origin of the thickness increase of the ZB segments is unclear. A more detailed discussion for an exemplary NW is conducted.

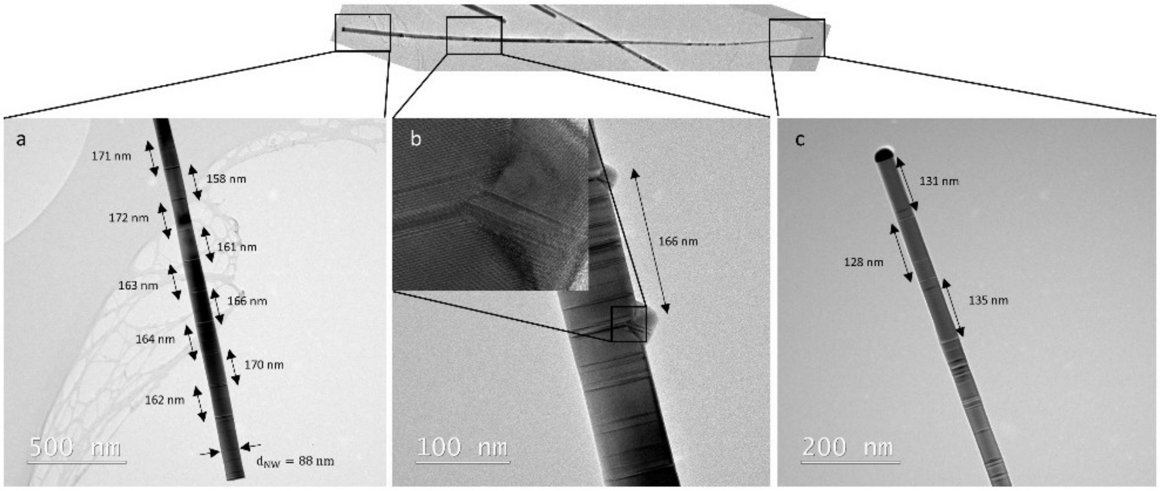

Figure S10: Exemplary TEM analysis of pulsed GaAs NW grown for 4.5 hr. The (a) bottom, (b) middle and (c) top of the NW are examined by TEM. (a) & (c) The crystal phase as well as the WZ segments length are studied. (b) GaAs shell growth around twinned ZB inclusions are shown.

An exemplary TEM analysis of an individual NW from bottom to top is shown in Figure S10. The WZ crystal phase is identified at the bottom of the NW in Figure S10a with equally spaced ZB inclusions. At a NW height of approximately 2.8 ± 0.5 µm, the formation of a multiple-twinned extension is observed. This lateral extension of the NW grows from a ZB inclusion. At this height along the NW, a region with a higher density of planar stacking faults is identified, which spans over approx. 600 nm. The precise origin of the increased density of stacking faults remains unclear. At the top of the NW, we observe the WZ phase with equally distributed ZB inclusions. A significant length decrease is observed, when comparing WZ segments from the bottom to the top of the NW. It is worth noting that we can not localize all ZB inclusions induced by the Ga-pulses from the pulse scheme depicted in Figure S1 in the analyzed NWs. The precise reason is unclear. We speculate that some ZB inclusions are overgrown by the pyramid-structure at the bottom of the NW. A more detailed analysis is beyond the scope of this work.

C. Growth rate analysis

The WZ segments between the ZB inclusions are used to analyze the growth rate of the examined NWs as a function of the NW length. The growth rates of seven NWs are presented from bottom to top in Figure S11. Initially, the growth rate varies between 50 and 70 nm/min. However, as the NW length increases, a general decline in growth rate is observed toward the top of the NW. The cause of this trend remains unclear but may be attributed to factors such as a decrease in adatom diffusion from the substrate, temperature variations (although simulations suggest only minor fluctuations),[1] or changes in the contact angle affecting the direct impingement collection area.

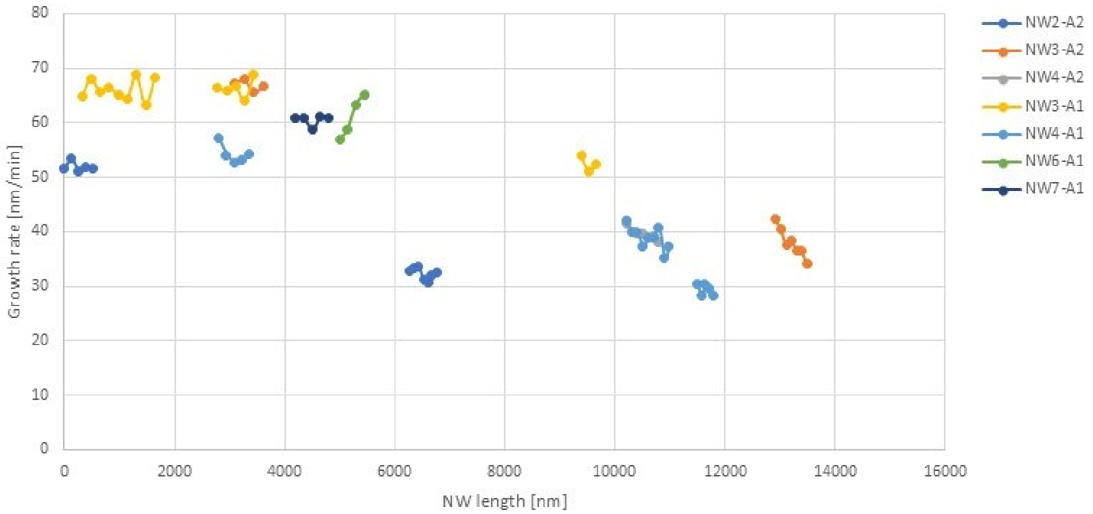

Figure S11: Growth rate analysis. The growth rate is examined by analyzing the WZ segments length divided by growth time from bottom to top of the NWs.

This observation suggests that further adjustments to the pulse scheme are necessary for longer WZ segment lengths. However, a precise determination of these adjustments is beyond the scope of this study.

D. Lower limit of Ga-pulsing duration

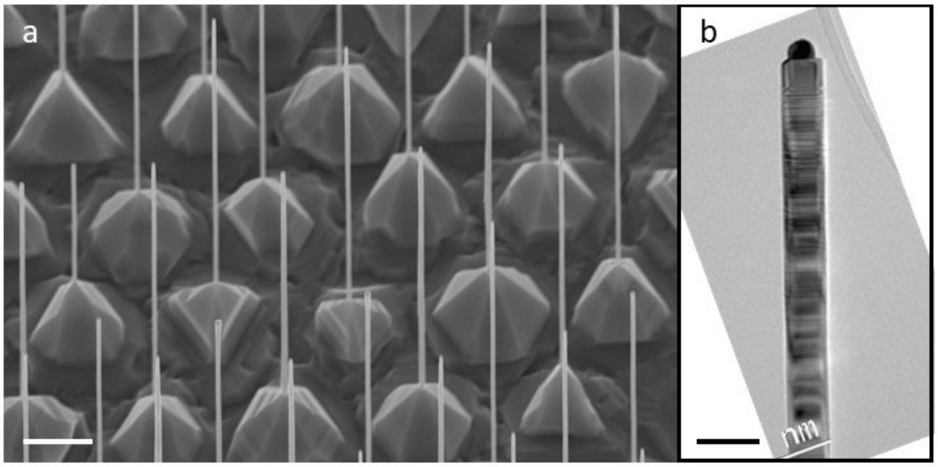

Figure S12: Lower limit of Ga-pulsed WZ GaAs NW growth. (a) NW array of pulsed NWs with a pulse duration of 6s. Scale bar is 1 µm. (b) Representative crystal phase evolution of NW top. Scale bar is 100 nm.

We attempted to minimize the ZB inclusion thickness in long hexagonal NWs by reducing the pulse duration from 7s to 6s. An SEM image of the growth attempt is shown in Figure S12a. However, TEM analysis showed a mixed phase segment at the top of the NWs (see S12b). We attribute this to an insufficient incorporation of Ga species into the catalyst particle with a Ga-pulse duration of 6 s. Based on these observations, we conclude that a 7s pulse duration is optimal for our growth conditions. Longer pulses were not explored to prevent an increase in ZB inclusion width.

5 Additional studies

A. Diameter dependency

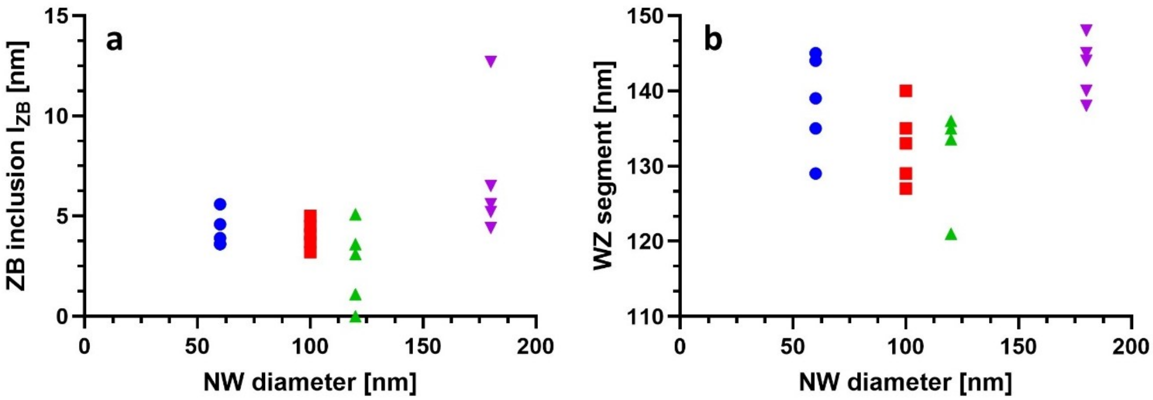

Figure S13: Diameter dependency of Ga-pulse. (a) ZB inclusion length as a function of NW diameter. (b) WZ segment length after Ga-pulse for varying NW diameter.

To evaluate the applicability of the pulsing scheme to different NW diameters, we investigated the effect of a Ga-pulse, $t_{pulse} = 10\ s$, on the ZB inclusion length and the subsequent WZ segment as a function of the NW diameter. We grew a sample with GaAs NW diameters ranging from 60 to 180 nm. After the Ga-pulse, NW growth continued for an additional 5 minutes. TEM studies revealed the following trends. The ZB inclusion length does not exhibit a clear dependency on the NW diameter (see Figure S13a). The growth rate of the WZ phase after the ZB inclusion also shows no distinct trend (see Figure S13b).
This observation aligns with previous studies, which reported no significant variation in growth rate as a function of NW diameter.[1,4] These findings indicate that the pulsing scheme can be extended to different diameters, demonstrating its flexibility and applicability across a broad diameter range.

B. V/III ratio study for prolonged wurtzite growth

In addition to the Ga-pulse study presented in the manuscript, we analyzed the effect of V/III ratio fine-tuning on the critical length. Therefore, we grew WZ GaAs NW stems with a V/III ratio of 2.4. Following the stem growth, the V/III ratio was slightly adjusted to 2.2, 2.3, and 2.5, respectively (see S14a). TEM analysis was used to examine the resulting crystal phases. As shown in Figure S14b, c, and d, the WZ phase was not maintained at the NW tips after these variations.

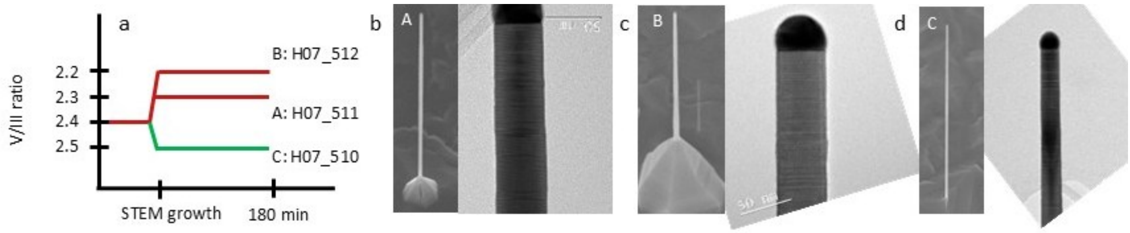

Figure S14. Influence of V/III ratio variations on critical length. (a) V/III study as a function of growth time. (b-d) SEM images and TEM images of the NW top's for a slight decrease in V/III ratio A, B as well as a slight decrease in C.

These results highlight the sensitivity of WZ phase stability to even minor changes in the V/III ratio, indicating that the optimal growth conditions are highly unstable. We therefore conclude that more precise adjustments—such as those enabled by Ga-pulses—are necessary to reliably stabilize the WZ phase.

References

1. Peeters, W. H. J. *et al.* Onset of uncontrolled polytypism during the Au-catalyzed growth of wurtzite GaAs nanowires. *Phys Rev Mater* **8**, 1–6 (2024).
2. Brown, E., Sheng, C., Shimamura, K., Shimojo, F. & Nakano, A. Enhanced charge recombination due to surfaces and twin defects in GaAs nanostructures. *J Appl Phys* **117**, (2015).
3. Isik Goktas, N., Sokolovskii, A., Dubrovskii, V. G. & Lapierre, R. R. Formation mechanism of twinning superlattices in doped gaas nanowires. *Nano Lett* **20**, 3344–3351 (2020).
4. Fadaly, E. M. T. *Epitaxy of Hexagonal SiGe Alloys for Light Emission*. (2021).
5. Rieger, T., Lepsa, M. I., Schapers, T. & Grützmacher, D. Controlled wurtzite inclusions in self-catalyzed zinc blende III-V semiconductor nanowires. *J Cryst Growth* **378**, 506–510 (2013).
